# Trafficking of Stretch-Regulated TRPV2 and TRPV4 Channels Inferred Through Interactomics

**DOI:** 10.3390/biom9120791

**Published:** 2019-11-27

**Authors:** Pau Doñate-Macián, Jennifer Enrich-Bengoa, Irene R. Dégano, David G. Quintana, Alex Perálvarez-Marín

**Affiliations:** 1 Biophysics Unit, Department of Biochemistry and Molecular Biology, School of Medicine, Universitat Autònoma de Barcelona, 08193 Cerdanyola del Vallés, Catalonia, Spain; pabdoama@gmail.com (P.D.-M.); jennifer.enrich@uab.cat (J.E.-B.); DavidG.Quintana@uab.cat (D.G.Q.); 2 Laboratory of Molecular Physiology, Department of Experimental and Health Sciences, Pompeu Fabra University, 08003 Barcelona, Catalonia, Spain; 3 Institut de Neurociències, Universitat Autònoma de Barcelona, 08193 Cerdanyola del Vallés, Catalonia, Spain; 4 CIBER Cardiovascular Diseases (CIBERCV), Instituto de Salud Carlos III, 28029 Madrid, Spain; iroman@imim.es; 5 REGICOR Study Group, Cardiovascular Epidemiology and Genetics Group, IMIM (Hospital Del Mar Medical Research Institute), 08003 Barcelona, Catalonia, Spain; 6 Faculty of Medicine, University of Vic-Central University of Catalonia (UVic-UCC), 08500 Vic, Spain

**Keywords:** ion channel trafficking, transient receptor potential channels, TRPV2, TRPV4, phosphatidylinositol signaling, stretch-related channels

## Abstract

Transient receptor potential cation channels are emerging as important physiological and therapeutic targets. Within the vanilloid subfamily, transient receptor potential vanilloid 2 (TRPV2) and 4 (TRPV4) are osmo- and mechanosensors becoming critical determinants in cell structure and activity. However, knowledge is scarce regarding how TRPV2 and TRPV4 are trafficked to the plasma membrane or specific organelles to undergo quality controls through processes such as biosynthesis, anterograde/retrograde trafficking, and recycling. This review lists and reviews a subset of protein–protein interactions from the TRPV2 and TRPV4 interactomes, which is related to trafficking processes such as lipid metabolism, phosphoinositide signaling, vesicle-mediated transport, and synaptic-related exocytosis. Identifying the protein and lipid players involved in trafficking will improve the knowledge on how these stretch-related channels reach specific cellular compartments.

## 1. Introduction

Transient receptor potential (TRP) channels are polymodal cation channels involved in somatosensation at the cellular and tissue levels in vertebrates [1]. Channels in the TRP family are in charge of sensing physical stimuli such as temperature or mechanical changes to trigger cation-mediated cell signal transduction pathways [2]. The vanilloid subfamily (TRPV) has six members (TRPV1–6), where TRPV1–4 have been long related to thermal sensing [3]. More recently, they have also been linked to mechanical stress, especially the TRPV2 and TRPV4 channels [4,5]. Accordingly, the function of TRPV2 and TRPV4 is expected to be essential in tissues with high mechanical shearing, such as skeletal and cardiac muscle. 

**Figure 1:**
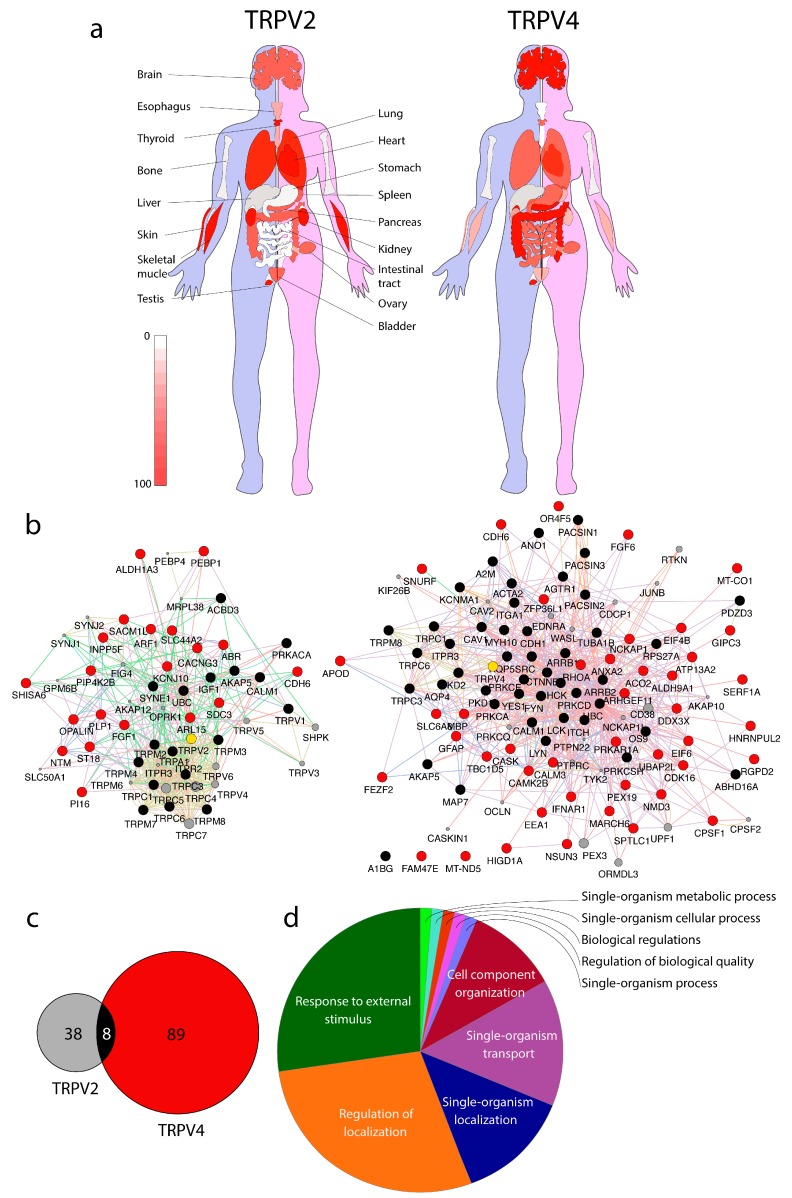
Transient receptor potential vanilloid subfamily TRPV2 and TRPV4 expression and interactomic profiles. (**a**) TRPV2 and TRPV4 tissue expression in humans; (**b**) TRPV2 (left) and TRPV4 (right) interactomes crossing published results (see main text for details). (**c**) Protein interactions overlap among TRPV2 and TRPV4 interactomes. (**d**) Main biological process terms defined by TRPV2 and TRPV4 gene set enrichment analysis (GSEA).

Expression of TRPV2 is wide (Figure 1a) and TRPV2 is involved in several physiological processes [6], but the particular role of this channel in skeletal and cardiac muscle is gaining much attention [7,8,9,10,11,12]. The expression of TRPV4 is as ubiquitous as TRPV2 (Figure 1a), but it is prominent in epithelial tissues, evoking calcium currents in response to extracellular stimuli, such as temperature, osmotic changes, and mechanical stretch [13,14,15]. Mutations of TRPV4 are related to involved in oligomerization, trafficking, and degradation can result in genetic disorders like Brachyolmia, Charcot–Marie–Tooth disease type 2C, spinal muscular spinal muscular atrophy, arthrogryposis, and hereditary motor and sensory neuropathy type 2 [16,17]. The identification of these mutations is a first step to determine the pathogenesis of the associated diseases and to design specific therapies. In addition, the disruption of the folding-sensitive region of TRPV4 could be a therapeutic option for diseases in which TRPV4 increases its activity such as pain and skeletal dysplasias [18]. Recently, it has also been shown that TRPV4 affects the calcium balance in cardiomyocytes, affecting contractility, leading to cardiac tissue damage in heart physiopathology [19].

An important question regarding TRPV2 and TRPV4 is how these channels are trafficked to and recycled from the membrane. This is an essential aspect in TRPV2 and TRPV4 function as Ca^2+^-dependent stretch-modulated channels. It has been shown that trafficking and/or translocation is a highly regulated process, and it may be dependent on channel activity. Translocation of TRPV2 to the membrane is driven by growth factors or chemotactic peptides [6], although is not clear yet whether TRPV2 is functional when at the plasma membrane and/or internal organelles, mainly because most the literature regarding TRPV2 trafficking is based on poor detection antibodies against TRPV2 [20]. The function of TRPV4 is exerted mainly at the plasma membrane and TRPV4 trafficking to the membrane is regulated by activators, such as GSK1016790A [18]. Controlled and regulated trafficking of ion channels, especially in excitatory tissues, is fundamental because of the possibility of cation leakage during trafficking, which leads to unbalanced cation homeostasis promoting cell toxicity and/or excitotoxicity. This review of the published protein–protein interactions for the TRPV2 and TRPV4 channels [21,22,23,24] intends to shed light about the proteins involved in the regulated and constitutive trafficking of these two mechanosensory cation channels.

## 2. Sequence and Structure Determinants in Channel Trafficking

Beyond TRP biogenesis and oligomerization as tetramers [25], TRP channel trafficking is a complex mechanism, integrating processes such as membrane insertion, glycosylation, Golgi maturation, vesicle trafficking, and protein–protein interaction (PPI) [26]. The balance between these processes results in a correct protein distribution in the plasma membrane or the corresponding membrane compartment, leading to up- and down-regulation of the protein function. The trafficking structural and molecular determinants in TRP sequence are mediators of PPI and/or lipid–protein interactions (LPIs). In TRPV channels, both the N- and C-termini were involved in trafficking. The distal N-terminus of TRPV channels is highly variable and structurally disordered and likely to host several phosphorylation sites and PPI and LPI domains. Deletion of the distal N-terminus of TRPV2 is enough to deplete the channel trafficking to the plasma membrane [23], which has also been observed for TRPV5 [27]. In addition, the N-terminus of TRPV4 interacts with OS-9 in the endoplasmic reticulum (ER), preventing channel trafficking to the plasma membrane [28]. Protein kinase C and casein kinase substrate in neurins proteins (PACSIN) also bind to the TRPV4 distal N-terminal domain (interaction exclusive for TRPV4 among TRPV channels), enhancing the relative amount of TRPV4 in the plasma membrane [29]. As for the C-terminus, AKAP79/150 binding has been described [24], as well as the ankyrin repeat domain (ARD), which is involved in PPI, but also in the complex mechanism of trafficking of TRPV channels. The TRPV4 C-terminus is also involved in trafficking, as shown by C-terminus deletion mutants, resulting in TRPV4 accumulation in the ER [30]. The characteristic ankyrin repeat domain (ARD) is involved in PPI, but also in the complex mechanism of trafficking of TRPV channels. Experiments carried out to map in vitro TRPV2 topology [23] were performed with constructs lacking either most of the N-terminal domain of the channel (the first 74 amino acids ΔN74-TRPV2) or the first 336 amino acids at the distal N-terminus (corresponding to the ARD, ΔARD-TRPV2). Despite the N-terminal truncation, TRPV2 was properly folded within the lipid bilayer. The ΔARD-TRPV2 mutant was not able to traffic to the plasma membrane, as determined by confocal imaging and biotinylation assay [23]. Such a fact indicates that the N-terminal region may be needed for additional channel processes other than insertion in the ER membranes, such as channel tetramerization, glycosylation, or interaction with chaperone proteins to allow membrane translocation. Although it has not been described for TRPV2 [31], the ARD indeed plays a key role in channel oligomerization for TRPV4, as has been shown for ARD mutants in TRPV4 [32]. TRPV mutants lacking ARD or carrying point mutations in N- or C-terminal domains produce channels that seem unable to tetramerize, which emphasizes the need for the TRPV cytosolic domains to promote the correct oligomerization of the subunits [32,33].

Turnover of TRPV channels in the plasma membrane is also controlled by regulated exocytosis, a process mediated by phosphorylation and interaction with SNAP (soluble N-ethylmaleimide sensitive fusion attachment protein) receptor (SNARE) complex proteins. SNAREs are a protein complex of more than 60 members in mammalian cells that mediate vesicle fusion with their target membrane bound compartment. The complex of SNAREs has a relevant role in cellular processes such as neurotransmitter release in synapses, exocytosis, or autophagy [34,35]. Upon protein kinase C (PKC) activation, TRPV1 is recruited to the plasma membrane by SNARE mediated vesicle transport, leading to a potentiation of TRPV1 currents [36]. Interaction of TRPV1 and TRPV2 with Snapin and SynaptotagminIX (SYT9) [36,37]. For TRPV2, the interaction with SNAREs protein is mapped into the highly conserved region of the membrane proximal domain (MPD), pointing to the conservation of these interactions along the TRPV1–4 subfamily [37].

Regarding LPI, TRPV channels share highly conserved PIP2 binding domains [24]. Binding of PIP2 to TRPV1 [38], TRPV2 [39], and TRPV3 [40] was mapped to the highly conserved proximal C-terminal domain, contiguous to the TRP box. The TRPV4 PIP2 binding domain is pinpointed using bioinformatics [24], although TRPV4 has an additional PIP2 binding site in the distal N-terminal domain [41]. Two recent works suggest that phosphatidic acid (PA) mediates LPIs between the membrane proximal domain (pre-S1 domain) and the C-terminus of TRPV channels in the trafficking of TRPV channels to the plasma membrane [37,42].

## 3. Interactomics

Proteomic studies provide useful tools to understand the molecular mechanism of TRPV channels. Guilt-by-association approaches aiming for mid-throughput interactome discovery for TRPV2 and TRPV4 were used, combined with standard techniques such as co-immunoprecipitation in mammalian cell lines and a novel membrane-specific yeast two-hybrid (MYTH) methodology [21,22].

The MYTH is a split-ubiquitin-based system that allows the location of the bait protein (TRPV2, TRPV4) embedded in the membrane. First, a yeast strain is generated that constitutively expresses the ion channel fused to the C-terminus of ubiquitin followed by the transcription factor LexA-VP16 (TF). The bait strain is then transformed with a cDNA library containing more than 10^6^ protein preys fused to the N-terminus of ubiquitin. The tag carries a mutation to hinder the spontaneous refolding of ubiquitin, which thus will only occur when a prey protein interacts with the bait. Once the interaction occurs and ubiquitin is refolded, the bait complex is cleaved and the TF is released. The traffic of TF to the nucleus starts the expression of reporter genes integrated in the yeast strain, allowing cells to grow in selective media lacking either Histidine (His) or Adenine (Ade). Moreover, the LacZ reporter gene is activated upon interaction, so that colonies carrying a putative interactor become blue stained in the presence of X-Gal. The blue color intensity may be used to determine whether interactions are strong or constitutive (intense blue color staining) or transient (pale blue color intensity) [43]. Using MYTH, 20 and 44 new interactors were found for TRPV2 and TRPV4, respectively [21,22] (Table S1 and Figure 1b,c).

Available PPIs for TRP channels are listed in the TRIP database [44]. For TRPV2 and TRPV4 PPI, refer to Supporting Table S1 and Figure 1b,c. The TRIP database is an excellent tool to get relevant information of biological processes regarding TRP channels. To the best of our knowledge, the database was last updated in August 2015, not including any new PPIs, such as the TRPV2 and TRPV4 membrane yeast two-hybrid (MYTH) dataset from these studies [21,24]. Figure 1 and Supplementary Table S1 list all the interactions for TRPV2 and TRPV4. However, for the PPI trafficking analysis performed in this study, only the PPIs identified in MYTH studies [21,22] are used. Using the list of TRPV2 and TRPV4 PPIs, a gene set enrichment analysis (GSEA) is performed, and then all ion channels and transporters filtered out to avoid the bias towards transport and cation transport gene ontology terms. Out of the 59 protein list, the GSEA resulted in 10 enriched biological process categories, including the following: neural nucleus development (GO:0048857); phosphatidylinositol biosynthetic process (GO:0006661); myelin sheath (GO:0043209); phospholipid biosynthetic process (GO:0008654); regulation of cellular amide metabolic process (GO:0034248); modulation of chemical synaptic transmission (GO:0050804); regulation of vesicle-mediated transport (GO:0060627); Ras GTPase binding (GO:0017016); regulation of cellular localization (GO:0060341); and cellular lipid metabolic process (GO:0044255). 

## 4. Understanding Channel Trafficking through Protein–Protein Interactions

In this review, the focus lies in terms related to two aspects: (i) lipid and phosphoinositides, and (ii) synaptic and vesicle-regulated trafficking. Because both aspects are tightly entangled, they and are difficult to study independently [21,22,23,24,37]. Lipids are key effectors in signal transduction and protein function, but also in protein trafficking [45]. Phosphoinositides (PIs) provide cellular organelles with specific lipid signatures, facilitating the binding of specific proteins. Membrane protein traffic is mediated by accessory proteins containing distinct lipid–protein binding domains, which promote binding depending on the lipids physico-chemical nature [45]. Regulated (e.g., synaptic-based exocytosis) and constitutive vesicle-mediated transport for TRP channels have already been reviewed, mostly based on TRPV1 studies [46]. This review aims to expand the knowledge on vesicle/synaptic-based trafficking by shedding some light on accessory proteins involved in these processes, as listed in Table 1.

**Figure 2:**
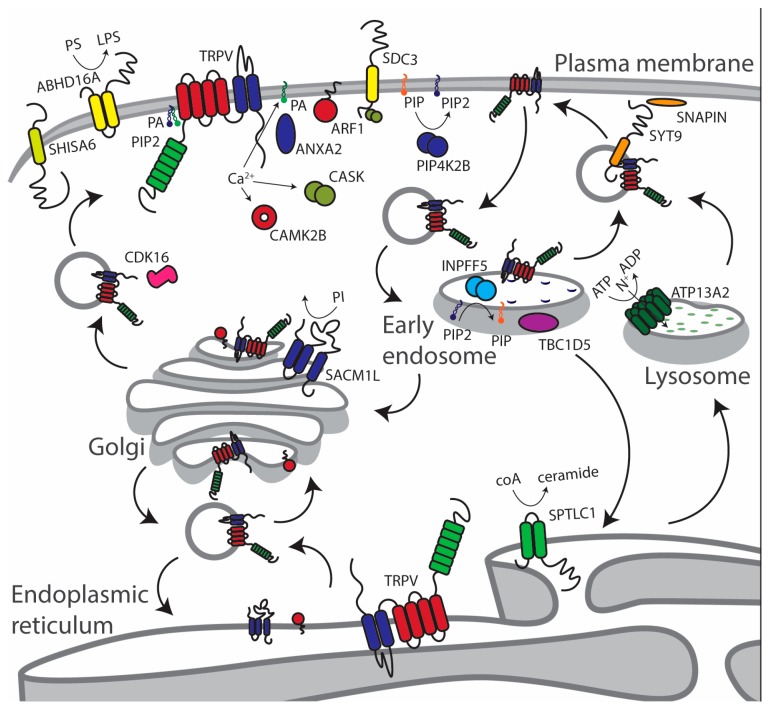
Cellular overview of the main TRPV2 and TRPV4 trafficking-related protein–protein interactions (PPIs) organized by cellular compartment (see main text for details).

Among the list of interactors (Table S1), the literature is revised regarding proteins/enzymes related to phosphoinositide signaling as markers of specific lipidic composition among cellular organelles, but also to trafficking and vesicle-mediated accessory proteins (Figure 2). This review provides an overview of the TRPV2 and TRPV4 trafficking process. Side information on TRPV2 and TRPV4 trafficking is found in the literature, such as the role of PACSIN3 for TRPV4 [29,41], recombinase gene activator (RGA) protein, and ras-related protein 7 (Rab7) for TRPV2 [47,48], and the Snapin/Syt9 pair for TRPV1 and TRPV2 [21,36,37]. However, the role of the lipid-mediated PPI for TRPV channels deserves extra attention, not only because of the complexity of the mechanism, but also because of the diversity of mechanisms depending on the tissue of interest (Figure 1a).

The lipase/acyl-transferase dehydrogenase enzyme (ABHD16A) is a TRPV4 interactor involved in lipid metabolism [49]. Proteins of the ABHD family are involved in lipidic modifications, such as palmitoylation, and in negative regulation of α-amino-3-hydroxy-5-methyl-4-isoxazolepropionic acid (AMPA) receptor trafficking. The depalmitoylase ABHD17A is crucial for synaptic targeting and vesicle sorting of AMPA receptors, through PSD-95 depalmitoylation [49,50]. In line with AMPA, but also to *N*-methyl-d-aspartate (NMDA) receptors trafficking, the calcium/calmodulin dependent protein kinase II beta (CAMK2B) and the calcium/calmodulin dependent serine protein kinase (CASK), both TRPV4 interactors, are trafficking regulatory proteins [51,52,53]. Another kinase interacting with TRPV4 is the cyclin dependent kinase 16 (CDK16), a key regulator of vesicle trafficking [54,55,56]. The kinase CDK16 interacts directly with COPII complexes modulating secretory cargo transport.

Annexin 2 (AnxA2) is a lipid raft associated trafficking factor in the plasma membrane and the endosomal system, related to both endo- and exocytosis [57]. Binding of AnxaA2 to phospholipids in a Ca^2+^-dependent manner [58], generating microdomains suitable for the binding of membrane proteins, such as the renal cotransporter NKCC2 [59]. AnxA2 has been shown to interact with fibroblast growth factor 1 (FGF1) (Table S1), forming heteroligomers capable of interacting with acidic membrane lipids, such as PA. Viral infection hijacks AnxA2, which is used for the virus advantage in cell-attachment, replication, and proliferation processes [60]; thus, AnxA2 is a likely candidate protein to play a role in the TRPV4-DDX3X mechanism in viral infectivity [22]. 

The ADP-ribosylation factor (Arf) small G proteins, such as Arf1, are related to lipid droplet metabolism, clathrin independent endocytosis, and other membrane dynamics processes [61,62,63]. N-terminus miristoylated Arf1 only tethers to the membrane when bound to guanosine triphosphate (GTP) [64]. Guanosine exchange factors (GEF) and GTPase activating proteins (GAP) are required by Arfs. Hydrolysis of GTP by Arf and Arf-like proteins regulate the enzymatic activity of proteins, such as PI kinases and phosphatases. Among the TRPV2 interactors, Phosphatidylinositol-5-phosphate 4-kinase type 2 beta (PIP4K2B), inositol polyphosphate-5-phosphatase F (INPP5F, also known as Sac2), and SAC1 like phosphatidylinositide phosphatase (SACM1L, also known as Sac1) are enzymes involved in phosphoinositide regulation as signals for membrane traffic. The Arf1 pathway is related to phospholipase D (PLD), which is responsible for the production of phosphatidic acid as a signaling molecule, shown to interact with TRPV channels [37,42]. The Arf1–PLD pathways are responsible for vesicle-mediated endocytosis and exocytosis, as well as the formation of multivesicular bodies (MVBs) through phosphoinositides binding/signaling [45]. The putative interaction between TRPV2 and Arf1 is an important hint regarding vesicle-mediated constitutive trafficking of TRPV channels. Other proteins that could be related to the Arf1 pathway, identified as TRPV2 and TRPV4 interactions, are Arl15 (Arf-like protein) and the CALM/CamK2B/Cask subset, respectively (Table S1 and Table 1).

Enzymes involved in phosphoinositide regulation are TRPV2/TRPV4 interactors, such as PIP4K2B and the SACM1L and INPP5F pair, Sac1 and Sac2, respectively. The kinase PIP4K2B regulates the levels of phosphatidylinositol 5-phosphate (PI5P) [65] by converting it to phosphatidylinositol 4,5-biphosphate (PI(4,5)P2), a lipid involved in TRPV2 and TRPV4 function [6,41]. The non-abundant PI5P phosphoinositide is related to the Akt kinase pathway and relevant for several cellular processes, such as survival and cell growth, with a prominent role in cancer [66]. Hydrolysis of phosphatidylinositol 3-phosphate (PI3P), phosphatidylinositol 4-phosphate (PI4P), and phosphatidylinositol 3,5-bisphosphate(PI(3,5)P2) is carried out by SACM1L/Sac1, which is enriched at the Golgi membrane, but is also present in ER membranes [67]. The preferential substrate for this enzyme is PI4P. The spatial distribution of PI4P in Golgi is defined by SACM1L/Sac1, creating the optimal conditions to maintain the cisternal identity of the Golgi, which is critical to membrane protein trafficking [68]. Inositol polyphosphate-5-phosphatase F (INPP5F, also known as Sac2) has a specific activity for PI(4,5)P2 and phosphatidylinositol 3,4,5-trisphosphate (PI(3,4,5)P3) to generate PI4P and PI(3,4)P2. INPP5F/Sac2 colocalizes with early endosomal markers and is related to the regulation of endocytic recycling [69].

The ATPase cation transporting 13A2 (ATP13A2 or PARK9) is an endo-/lysosomal associated ATPase related to intracellular trafficking under proteotoxic stress [70]. In a MYTH-based proteomics approach [71], the authors have identified 43 PARK9-interacting proteins related to trafficking. The putative TRPV4-PARK9 and the TRPV2-INPP5F interactions identified in studies [21,22] could be interesting to understand TRPV channels recycling in the endosome/lysosome pathways.

Syndecan-3 (SDC3) is a heparan-sulfate proteoglycan and a TRPV2 interactor, related to leukocyte migration [72]. Modification of cytoskeleton is carried out by SDC3, but no relationship between SDC3 and membrane protein trafficking has been shown so far. However, SDC4 modulates the activity and membrane expression of TRPC6 in glomerular permeabilization [73].

The trafficking of AMPA receptors towards the synapse has been thoroughly studied [74], and among the proteins involved in locating AMPA receptors at the synapse, SHISA6 is found as a TRPV2 interactor. The C-terminal characteristic PDZ domain in SHISA6 binds to post-synaptic density protein 95 (PSD95), which confines AMPA receptors at the postsynaptic density [75]. Proteins, such as SHISA6, ABHD16A, Snapin, and SYT9 (Snapin and SYT9 revised earlier), interact with TRPV channels, mediating synaptic exocytosis, suggesting conservation of some elements in regulated exocytosis of TRPV channels [21,36,37,46].

The TRPV4 interactor serine palmitoyltransferase long chain base subunit 1 (SPTLC1), which resides in the ER, drives the synthesis of sphingomyelin [76], sphingolipid that needs to be trafficked to the plasma membrane. Specific lipid composition in membrane domains may argue for the need of specific lipids bound to TRPV channels during trafficking. SPTLC1, teaming up with ORMDL3, are involved in ceramide synthesis [77,78], in response to ER stress and calcium homeostasis, factors influencing the trafficking of membrane proteins, such as TRPV channels.

The TBC1 domain family member 5 (TBC1D5) plays a significant role in the regulation of the endosomal pathway. TBC1D5 interacts with TRPV4 in a yeast two-hybrid approach [22]. In the endosomal system, TBC1D5 inhibits the retromer and promoting autophagy. It is also a key factor in membrane turnover and membrane protein recycling through Rab7 [79]. The Rab7 and TRPV2 pair has been shown to colocalize in the endosome. As a result of such a trafficking situation, TRPV2 relates to nervous system development by enhancing neurite outgrowth [48]. Among TRPV channels, TRPV2 is the most likely to play a role in the regulation of the endosomal pathway [80]. 

## 5. Concluding Remarks

This review of the literature aims toward a better understanding of the mechanisms driving the trafficking of TRPV2 and TRPV4 to specific membrane compartments, derived from a guilt-by-association approach based on PPI (Table S1). The interaction of TRPV2 and TRPV4 with lipids and/or with lipid modifying enzymes points to the fact that the lipid environment is fundamental for TRPV2 and TRPV4 trafficking, beyond the TRPV2 and TRPV4 lipid requirement for ion channel function. Future perspectives should include the study of the tight inactivation mechanisms of ion channels involved in the trafficking of these proteins, to prevent cation leakage inside the cell. In the case of TRPV2 and TRPV4 (and other TRP channels), the focus so far has resided on which lipid signaling molecules activate/inhibit the channel at the site of function. The identification of LPI and PPI interactions preventing cation leakage during trafficking toward avoiding detrimental cell toxicity effects provides another potentially interesting area of study, which may be especially relevant in tissues under high stretch stress conditions, such as skeletal and cardiac muscle. Knowledge on how TRPV2 and TRPV4 are specifically trafficked in these tissues might provide invaluable benefits in the therapeutic management of muscle physiopathology, where cation transport and balance play a cardinal role.

## Figures and Tables

**Table 1:** List of interactors related to trafficking processes derived from a membrane yeast two hybrid approach.

Gene Symbol	Interactor	Gene Name	Gene ID	Process ^a^
*ABHD16A*	TRPV4	abhydrolase domain containing 16A	7920	1
*ANXA2*	TRPV4	annexin A2	302	1, 2
*ARF1*	TRPV2	Adenosine diphosphate (ADP) ribosylation factor 1	375	1, 2, 3, 4, 5
*ATP13A2*	TRPV4	Adenosine triphosphate (ATP)ase cation transporting 13A2	23400	2,3
*CAMK2B*	TRPV4	calcium/calmodulin dependent protein kinase II beta	816	4
*CASK*	TRPV4	calcium/calmodulin dependent serine protein kinase	8573	2,3,4
*CDK16*	TRPV4	cyclin dependent kinase 16	5127	2
*INPP5F*	TRPV2	inositol polyphosphate-5-phosphatase F	22876	1, 2, 3, 5, 6
*PIP4K2B*	TRPV2	phosphatidylinositol-5-phosphate 4-kinase type 2 beta	8396	1, 5, 6
*SACM1L*	TRPV2	SAC1 like phosphatidylinositide phosphatase	22908	1, 5, 6
*SDC3*	TRPV2	syndecan 3	9672	1, 2, 3, 4, 5
*SHISA6*	TRPV2	shisa family member 6	388336	2, 4
*SNAPIN*	TRPV2/TRPV1	SNAP associated protein	23557	2, 3, 4
*SPTLC1*	TRPV4	serine palmitoyltransferase long chain base subunit 1	10558	1, 6
*SYT9*	TRPV2/TRPV1	synaptotagmin 9	143425	2, 3, 4
*TBC1D5*	TRPV4	TBC1 domain family member 5	9779	3

^a^ Processes: 1, cellular lipid metabolism; 2, regulation of cell localization; 3, regulation of vesicle-mediated transport; 4, modulation of chemical synaptic transmission; 5, phosphatidylinositol biosynthesis; 6, phospholipid biosynthesis. TRPV, transient receptor potential vanilloid subfamily; SNAP, soluble NSF attachment protein.

## Supplementary Materials

The following are available online at https://www.mdpi.com/2218-273X/9/12/791/s1, Table S1. TRPV2 and TRPV4 interactomes.
